# Rare Implantation Sites of Ectopic Pregnancy: A Case Series of Ovarian and Hepatic Pregnancy and Review of Diagnostic Challenges

**DOI:** 10.3390/clinpract16060107

**Published:** 2026-05-31

**Authors:** Stefan Ivanovic, Ljubomir Srbinovic, Milica Ivanovic, Dragana Maglic, Nenad Kokošar, Rastko Maglic

**Affiliations:** 1 Obstetrics and Gynecology Clinic “Narodni Front”, 11000 Belgrade, Serbia; 2 Faculty of Medicine, University of Belgrade, 11000 Belgrade, Serbia

**Keywords:** ectopic pregnancy, ovarian pregnancy, hepatic pregnancy, abdominal ectopic pregnancy, diagnostic challenges, laparoscopy, case series

## Abstract

**Background:** Ectopic pregnancy remains a significant cause of maternal morbidity in early pregnancy. While most ectopic pregnancies happen within the fallopian tube, implantation may rarely occur in atypical locations such as the ovary or abdominal cavity. These rare forms often present with nonspecific clinical findings and may represent a considerable diagnostic challenge. **Methods:** We report a case series of three rare ectopic pregnancies managed at a tertiary referral center. Two cases involved ovarian pregnancy, and one case represented an exceptionally rare hepatic ectopic pregnancy. Clinical presentation, diagnostic pathway, surgical management, and outcomes were analyzed and compared with available literature. **Results:** In the first two cases, ovarian pregnancy was confirmed intraoperatively and treated surgically, with ovarian preservation in one patient and adnexectomy in the other due to active bleeding. The third case had an unusual course: initial surgery was performed for hemoperitoneum caused by a ruptured corpus luteum cyst, while persistent β-hCG elevation later led to identification of hepatic ectopic pregnancy, confirmed by imaging and surgery. All patients recovered favorably, with complete β-hCG negativization. **Conclusions:** Rare ectopic implantation sites may mimic acute abdominal conditions and remain difficult to diagnose preoperatively. High clinical suspicion, serial β-hCG monitoring, and appropriate imaging are essential. Surgical management remains central, particularly in life-threatening bleeding. Standard algorithms for tubal ectopic pregnancy may not be fully applicable and should be adapted to the clinical context.

## 1. Introduction

Ectopic pregnancy (EP) refers to implantation of the blastocyst outside the endometrial cavity and remains one of the leading causes of maternal morbidity in the first trimester. Its estimated incidence is up to 2% of all pregnancies [1]. Although most EPs occur in the fallopian tube (up to 95%), implantation may also occur at less common sites, including the ovary (accounting for approximately 3% of EPs), the cervix, the interstitial portion of the fallopian tube, a cesarean section scar, or within the abdominal cavity [2,3]. Although these implantation sites are uncommon, they represent a considerable diagnostic and therapeutic challenge because they frequently present with atypical clinical findings and may lead to severe, potentially life-threatening complications.

Ovarian pregnancy (OP), defined as implantation of the gestational sac within the ovarian parenchyma, represents a rare form of EP, accounting for approximately 0.5–3% of all EPs (estimated incidence 1/2100–1/40,000 pregnancies), most commonly occurring on or in close proximity to the corpus luteum and often clinically or sonographically mimicking ruptured or hemorrhagic luteal cysts. First described in the 17th century, its modern diagnostic definition was later formalized through the Spiegelberg criteria [4]. This rare clinical entity poses a significant diagnostic challenge, as its clinical presentation often overlaps with more common acute gynecological conditions, such as rupture of the corpus luteum or hemorrhagic and endometriotic cysts. Patients most frequently present with nonspecific lower abdominal or pelvic pain, with or without signs of hemodynamic instability [5]. Despite advances in diagnostic techniques and the widespread availability of transvaginal ultrasonography (TVUS), establishing a preoperative diagnosis of ovarian pregnancy remains difficult in routine clinical practice.

Hepatic pregnancy (HP) represents an exceptionally rare form of abdominal EP, with an estimated incidence of approximately 1% of all EPs [6]. Implantation of gestational tissue within the hepatic parenchyma carries a high risk of massive intra-abdominal hemorrhage, while early symptoms are often absent or nonspecific, making timely diagnosis particularly challenging [6]. Ultrasonography (US) may fail to detect this localization, and the role of medical management with methotrexate (MTX) is limited, as hepatic HPs are most often identified at an advanced stage [7]. Because of its extreme rarity and nonspecific clinical presentation, the condition may remain unrecognized until serious complications develop. TVUS combined with measurement of serum β-hCG remains the cornerstone of the diagnostic approach. Definitive confirmation of the diagnosis is frequently made intraoperatively [7]. Owing to the rarity of these conditions, many cases are identified only during surgical intervention, and the true incidence is likely underestimated. Delayed diagnosis is associated with an increased risk of life-threatening intra-abdominal hemorrhage, the need for more radical surgical procedures, and potential loss of reproductive potential. To date, the available literature addressing ovarian and abdominal EP remains limited and largely consists of isolated case reports and small case series [8]. Current evidence often provides limited guidance regarding the optimal diagnostic algorithm and the most appropriate surgical management for these unusual implantation sites. Given their rarity and nonspecific clinical presentation, clinicians may encounter such forms of EP only once or a few times during their professional careers, which further complicates timely recognition and treatment [8].

The aim of this case series is to present rare cases of OP and HP managed at our center and diagnosed intraoperatively, with particular emphasis on the diagnostic challenges, surgical management, and clinical relevance of these unusual implantation sites. We underscore the importance of including them in the differential diagnosis of acute pelvic pain and early pregnancy complications, given that standard diagnostic and therapeutic algorithms developed for tubal EPs cannot be reliably applied to these rare locations.

## 2. Case Presentations

### 2.1. Case I

A 28-year-old woman (G0P0) was admitted due to secondary amenorrhea, abnormal β-hCG dynamics, and a suspicious US finding in the region of the right adnexa. The duration of amenorrhea was six weeks. Her last menstrual period occurred on time and was of usual character and normal duration. Her medical history was notable for hypothyroidism treated with levothyroxine 50 µg once daily and insulin resistance treated with metformin XR 750 mg once daily. She was a non-smoker. Several months earlier, the patient had a documented *Mycoplasma genitalium* infection. According to the patient’s medical history, a follow-up PCR swab performed after treatment was negative. She denied previous use of an intrauterine device (IUD) as well as any prior EP.

On examination, the patient was afebrile, normotensive, eupneic, and in good general condition, without subjective complaints. The abdomen was soft and non-tender on palpation. Speculum examination revealed no vaginal bleeding. Bimanual pelvic examination demonstrated a uterus of normal size, firm and mobile, positioned in retroversion and retroflexion, while the adnexa were free and non-tender on palpation.

TVUS demonstrated a retroverted and retroflexed uterus with an endometrial thickness of 11 mm and no visible intrauterine gestational sac. In the region of the right adnexa, a suspicious mass measuring 51 × 37 mm was identified. The left adnexa appeared normal, and no free fluid was detected in the pouch of Douglas. Laboratory findings at admission (Table S1) showed no significant abnormalities, except for abnormal β-hCG dynamics. At admission, the patient provided her most recent previous β-hCG value of 1134 mIU/mL, with information that earlier values measured over the preceding several days had been at a similar level. The β-hCG value measured at our institution on admission was 1098 mIU/mL, while a repeated measurement after 24 h showed a value of 1197 mIU/mL. The patient subsequently underwent laparoscopic surgery. Intraoperatively, both fallopian tubes were found to be completely intact, normal in appearance, and freely mobile. However, an ovarian EP was identified on the right ovary (Figure 1). A laparoscopic partial oophorectomy was performed (Figure 2). Histopathological examination confirmed the diagnosis of ovarian EP. The postoperative course was uneventful, and the patient was discharged 24 h after surgery (total hospital stay: 3 days) in stable general condition, afebrile and normotensive. During further outpatient follow-up, serial serum β-hCG measurements were performed until complete negativization.

### 2.2. Case II

A 36-year-old woman (G3P2) was admitted due to lower abdominal pain following a six-week period of amenorrhea. Her reproductive history included two deliveries by cesarean section and one spontaneous miscarriage. The patient reported smoking up to 20 cigarettes per day. She denied a history of pelvic inflammatory disease (PID), previous IUD use, or prior EP.

Upon admission, the abdominal pain had resolved spontaneously. On clinical examination, the patient was afebrile and normotensive. Abdominal palpation revealed no remarkable findings. There was no vaginal bleeding. Gynecological examination demonstrated a mildly enlarged and slightly softened uterus. In the region of the right adnexa, a firm, mobile, and mildly tender adnexal mass measuring approximately 5 cm was palpated.

Laboratory findings at admission (presented in Table S2) showed a β-hCG level of 18,773 mIU/mL and hemoglobin of 117 g/L. TVUS demonstrated an enlarged uterus measuring 98 × 65 × 61 mm with an endometrial thickness of 13 mm and no visualized intrauterine gestational sac. In the region of the right adnexa, a hyperechoic structure measuring 62 × 41 mm was identified, containing a central anechoic round formation measuring 16 mm in diameter, suspicious for EP (Figure 3). The left adnexa appeared normal. A small amount of free fluid was noted in the pouch of Douglas. Laparoscopic surgery was performed. Intraoperatively, the right adnexa were covered with clotted blood. Further exploration revealed an enlarged right ovary (~5 cm) with a ruptured OP and active bleeding. Approximately 300 mL of partially clotted and liquid blood was present in the peritoneal cavity. Due to persistent bleeding and the inability to achieve adequate hemostasis after partial oophorectomy, a right adnexectomy was performed. The left adnexa appeared normal. The estimated total blood loss was 400 mL. Histopathological examination confirmed the diagnosis of OP. During the postoperative course, the patient developed anemia, which was corrected with blood transfusion. The patient was discharged 48 h after surgery (total hospital stay: 3 days) in stable general condition, afebrile and normotensive. During further outpatient follow-up, serial serum β-hCG measurements were performed until complete negativization.

### 2.3. Case III

A 40-year-old woman (G0P0) was admitted to the clinic due to lower abdominal pain, weakness, and fatigue following a six-week period of amenorrhea. In her medical history, the patient reported treatment for infertility and stated that five years earlier she had undergone surgery for endometriosis, when a right ovarian cystectomy had been performed through a lower transverse laparotomy (Pfannenstiel incision). She was a non-smoker and denied any previous PID or IUD use. Laboratory findings on admission are presented in Table S3.

The initial serum β-hCG level on admission was 621 mIU/mL. At admission the patient appeared weak and exhausted, with cold sweating. Blood pressure was 90/70 mmHg and pulse rate 130/min. A peripheral venous line was immediately established, resuscitation measures were initiated, and a urinary catheter was placed. Urine output was less than 30 mL per hour. US examination demonstrated free intraperitoneal fluid in the pelvis and abdomen. TVUS showed normal-appearing adnexa bilaterally, and no gestational sac was visualized within the uterine cavity. Due to clinical signs of intra-abdominal bleeding and hypovolemic shock, an emergency surgical procedure was performed. Intraoperatively, rupture of a corpus luteum cyst of the left ovary was identified, accompanied by massive intra-abdominal bleeding exceeding two liters. Both fallopian tubes and the right ovary appeared normal. The small bowel, cecum, rectosigmoid colon, and infracolicomentum were carefully inspected, with no evidence of EP. Histopathological analysis confirmed a corpus luteum cyst of the left ovary, suggesting that the pregnancy remained of unknown location.

Postoperatively, the patient recovered clinically; however, β-hCG levels continued to rise, reaching 1052 mIU/mL. Follow-up US examination showed no evidence of intrauterine or EP. A small hepatic lesion measuring 37 × 25 mm was noted during US examination but was initially considered clinically insignificant (Figure 4). On the third postoperative day, diagnostic dilatation and curettage of the uterine cavity was performed. Histopathological examination demonstrated endometrial intraepithelial neoplasia, without evidence of intrauterine pregnancy. Following this intervention, β-hCG levels decreased to 861 mIU/mL but subsequently rose again to 1132 mIU/mL. As intrauterine pregnancy had been excluded, abdominal EP of unknown localization was suspected, and a single dose of MTX (50 mg) was administered. Three days later, the follow-up β-hCG level increased to 1668 mIU/mL. Five days after MTX administration, the patient again developed pain, now localized in the upper abdomen. The β-hCG level at that time was 2060 mIU/mL. US examination demonstrated a small amount of free intraperitoneal fluid and an abnormal appearance of the liver, with a hyperechoic mass measuring approximately 6 cm in diameter in the left hepatic lobe near the hepatic hilum. On physical examination, tenderness in the upper abdomen was present. These findings raised suspicion of an EP localized in the liver. The patient was subsequently transferred to the abdominal surgery clinic for further evaluation and management. Urgent computed tomography (CT) demonstrated a mass measuring 6 × 5 cm in the left hepatic lobe (between segments III and IV), with a central oval area of low density and a peripheral area of higher density following contrast enhancement (Figure 5). A significant amount of fluid with blood density was detected beneath the left hepatic lobe and above the stomach. Following the urgent CT examination, emergency surgery was performed via an upper midline laparotomy. Approximately 500 mL of predominantly clotted blood was found between the left hepatic lobe and the stomach. After evacuation of the blood, an EP was identified beneath the falciform ligament in hepatic segment III (Figure 6). The EP was removed, and the cavity was packed with gauze soaked in hypertonic sodium chloride solution. More than three liters of isotonic sodium chloride solution were instilled into the abdominal cavity, and two drains were placed—one in the subhepatic space and the other in the pouch of Douglas. Serum β-hCG levels decreased to 460 mIU/mL on the first postoperative day. Six days after surgery, the β-hCG level was 32 mIU/mL. The initial hemoglobin value did not reflect the severity of the clinical condition, which was consistent with early hypovolemic shock before laboratory changes became apparent. Preoperative hemoglobin was 109 g/L, and at discharge it was 110 g/L following a single postoperative transfusion of 280 mL of blood. The patient was discharged, seventh postoperative day (total hospital stay: 7 days), in good general condition. During further outpatient follow-up, serial serum β-hCG measurements were performed until complete negativization. Histopathological examination confirmed EP with trophoblastic tissue present within the liver. This case has been previously reported from our institution [9] and is included here within a case series to provide additional clinical context and comparative analysis.

Comparative summary of clinical characteristics, diagnostic findings, and management of the three presented cases are present in Table 1.

## 3. Discussion

These cases illustrate the diagnostic complexity particularly when implantations occur at unusual anatomical sites. Although most EPs occur within the fallopian tube, non-tubal implantations such as ovarian and abdominal pregnancies represent distinct clinical entities that may lead to severe complications if not recognized promptly. Over the past decades, an increase in the reported incidence of ovarian pregnancy has been observed, reflecting both improved diagnostic capabilities and broader use of assisted reproductive technologies (ART). Several risk factors have been associated with ovarian pregnancy, including the use IUD, ART, prior abdominal or pelvic surgery, pelvic adhesions, and disturbances of ovulation [3,8]. In women using IUDs, possible mechanisms include alterations in tubal motility due to hormonal influences as well as changes in cervical mucus composition that may interfere with normal embryo transport. In the setting of ART, implantation of a fertilized ovum into a puncture site created during oocyte retrieval or into scar tissue formed during previous ovulatory cycles has been proposed as a potential mechanism [10]. Furthermore, medications used during controlled ovarian stimulation may influence tubal motility and embryo transport, potentially contributing to abnormal implantation sites [11]. Despite major advances in imaging modalities and the widespread availability of high-resolution TVUS, the clinical presentation of OP remains challenging. Delayed recognition is associated with a substantial risk of rupture, massive intra-abdominal hemorrhage, hemorrhagic shock, and the need for urgent surgical intervention, all of which may lead to severe morbidity or even mortality if not managed promptly [8]. Reports from the literature describe implantation of the blastocyst most frequently at the site of a recently ruptured follicle, within an ovulatory scar, or in ovarian tissue altered by previous surgical procedures [12,13]. In contrast to OP, hepatic implantation represents an exceptionally rare form of abdominal EP. The most widely accepted mechanism involves transperitoneal migration of the fertilized ovum following tubal abortion or fimbrial escape, after which the blastocyst attaches to highly vascularized hepatic surfaces, most commonly beneath the falciform ligament [13]. Pre-existing micro-injuries, inflammatory processes, or focal hepatic adhesions may facilitate implantation at this site [13]. Because of the extreme vascularity of hepatic tissue, such implantations carry a particularly high risk of catastrophic intra-abdominal bleeding.

### 3.1. Clinical and Biochemical Presentation

#### 3.1.1. Ovarian Ectopic Pregnancy

The clinical presentation of OP is frequently nonspecific and may vary considerably among patients. The most reported symptoms include amenorrhea, lower abdominal pain, and vaginal bleeding of varying intensity, although in certain cases symptoms may be mild or entirely absent [14]. This variability in clinical manifestations contributes significantly to diagnostic delay. US findings are similarly nonspecific. Typical US features include an enlarged ovary containing a peripheral vascularized lesion known as the “ring of fire” sign, often surrounding a central anechoic area [14]. Differentiation from other ovarian cystic structures, particularly corpus luteum or hemorrhagic cysts, may be difficult. Several authors have emphasized that classical clinical signs of EP may be absent, a finding that was also observed in the cases presented in our series [14,15]. Surgical excision remains the treatment of choice in most cases. Caution is required in heterotopic pregnancies, most commonly occurring after ART procedures, where preservation of the corpus luteum and avoidance of uterine manipulation are essential [16]. Recent reports have further demonstrated that ART procedures may be associated with extremely rare implantation sites and delayed diagnosis, emphasizing the importance of maintaining a high index of suspicion in this patient population [17]. In an analysis of 112 ovarian pregnancies by Shao et al., the most frequently identified risk factors were previous abdominal surgery and the use of IUDs. The rupture rate in this cohort was high, reaching 86.6%, and all patients required surgical treatment either by laparotomy or laparoscopy [18]. Laparoscopic management was associated with shorter hospitalization, faster postoperative recovery, and a lower rate of postoperative fever, without significant differences in operative duration or blood loss. Consequently, whenever feasible, laparoscopy should be considered the first-line surgical approach [19]. Notably, in our two ovarian pregnancy cases no recognized risk factors were identified. Ren et al. described two cases of unruptured ovarian pregnancy presenting with the classical triad: amenorrhea, pelvic pain, and vaginal bleeding. In these patients, sequential β-hCG measurements, repeated TVUS examinations, and when necessary, MRI significantly improved diagnostic accuracy [14,15]. Treatment consisted of laparoscopic excision of the intact gestational sac with maximal preservation of ovarian tissue. OP may also present as an acute surgical emergency due to rupture and hemoperitoneum, as illustrated by Qing et al. In that report, the patient presented with severe abdominal pain, declining hemoglobin levels, β-hCG concentrations of approximately 1800 mIU/mL, and a cystic adnexal lesion accompanied by free intraperitoneal fluid. Surgical excision of the ovarian lesion with local MTX administration was performed, and the diagnosis was confirmed histopathologically [20]. The severity of this condition is further reflected in our second case, where massive bleeding and the inability to achieve adequate hemostasis necessitated adnexectomy. Because symptoms and imaging findings are often nonspecific, OP may not initially be considered in differential diagnosis, particularly in hemodynamically stable patients without clear signs of intra-abdominal bleeding. In such circumstances, surgical management remains the gold standard, whereas systemic MTX therapy demonstrates lower effectiveness compared with tubal EP [20].

#### 3.1.2. Hepatic Ectopic Pregnancy

Ectopic implantation within hepatic tissue is exceedingly rare but carries a substantial risk of catastrophic hemorrhage [21]. For this reason, individual case reports remain an important source of clinical information for physicians who may encounter this condition only once in their careers. US, contrast-enhanced computed tomography demonstrating characteristic peripheral enhancement with central hypodensity, and FDG PET-CT showing peripheral metabolic uptake may all contribute to accurate localization of the EP [21]. CT clearly demonstrated a hepatic lesion suspicious for EP, allowing prompt surgical intervention. Katiyar et al. reported that HP may mimic gastrointestinal or hepatobiliary disease, which frequently leads to delayed diagnosis and increased risk of intraperitoneal hemorrhage [22]. US is usually the initial imaging modality, while CT and MRI provide additional anatomical detail and assist in surgical planning. Surgical treatment remains the mainstay of therapy and may involve laparoscopy or laparotomy, with possible segmental liver resection when required [22,23]. Additional therapeutic approaches including MTX therapy, hepatic artery ligation, vascular embolization, or monopolar coagulation have been described in selected cases [13,22]. When placental tissue is present, management must be individualized because removal of the placenta may result in uncontrollable bleeding [24]. In some situations, the placenta is intentionally left in situ, as demonstrated by Febriastuti et al., who reported successful management of a viable HP at six months’ gestation by removing the fetus and leaving the placenta in place after MTX administration [25]. Our third case demonstrates several features frequently described in the literature, including nonspecific symptomatology, potential for sudden hemodynamic deterioration, and the crucial role of advanced imaging following initial US evaluation. As expected, removal of trophoblastic tissue was followed by a rapid postoperative decline in β-hCG levels [13,22]. HPs have been reported in different liver segments, most frequently beneath the falciform ligament [13]. Clinical severity largely depends on gestational age. Early pregnancies may remain asymptomatic or present with mild nonspecific symptoms, whereas later gestations may manifest with severe abdominal pain, acute abdomen, or hemorrhagic shock [26]. Because of the extreme vascularity of hepatic tissue and the associated risk of massive bleeding, surgical management is often complex and requires a multidisciplinary team including hepatobiliary surgeons [26].

### 3.2. Diagnosis

Serial monitoring of serum β-hCG levels combined with US examination, both transvaginal and transabdominal, represents the initial diagnostic approach [27]. The detection of free intraperitoneal fluid on TVUS may represent an important predictor of clinical urgency and hemodynamic instability, particularly in atypical or ruptured EPs [28]. In cases with atypical clinical presentation or inconclusive US findings, instrumental revision of the uterine cavity with histopathological analysis may be necessary [29]. If β-hCG levels fail to decline adequately following such intervention, further diagnostic evaluation should include advanced imaging techniques such as CT or MRI to identify the site of implantation and facilitate surgical planning [30]. This step is particularly important in suspected hepatic HP because of the need to differentiate this condition from other hepatobiliary pathologies. Definitive diagnosis is established by histopathological confirmation according to Spiegelberg’s criteria, which require an intact fallopian tube, a gestational sac located on the ovary, attachment of the ovarian ligament to the gestational sac, and histological demonstration of trophoblastic tissue within ovarian tissue [31]. Postoperative monitoring of β-hCG levels remains essential to confirm complete removal of trophoblastic tissue and to detect persistent disease at an early stage.

### 3.3. Management

Surgical treatment remains the predominant therapeutic approach in OP and may be performed either by laparoscopy or laparotomy, depending on the patient’s clinical condition, the feasibility of laparoscopic access, and the surgeon’s experience [13,18]. Systemic MTX therapy has been associated with relatively low success rates in this setting [18]. Similarly, HP is primarily managed surgically. Intraoperative assessment plays a crucial role in determining the most appropriate surgical strategy, including whether removal of the placenta should be performed immediately or postponed as a secondary procedure [13]. Careful postoperative monitoring is required, with particular attention to hemodynamic stability and the progressive decline of serum β-hCG levels [28]. Whenever possible, fertility-preserving surgical techniques are preferred because they support the patient’s future reproductive potential [32]. Laparoscopic procedures, when feasible, offer several advantages including shorter hospitalization, faster postoperative recovery, and reduced morbidity compared with more invasive surgical approaches [33]. In hemodynamically unstable patients or in settings with limited resources, laparotomy remains the preferred approach to achieve rapid control of hemorrhage [34].

### 3.4. Clinical Implications

The presented cases highlight several important clinical considerations. OP remains an uncommon diagnosis that may easily be overlooked in the differential evaluation of early pregnancy complications. Histopathological confirmation therefore continues to represent the diagnostic gold standard. In abdominal EPs, advanced imaging techniques such as CT and MRI are essential for precise localization of the implantation site and for planning the optimal surgical approach. A multidisciplinary strategy involving gynecologists, radiologists, and hepatobiliary surgeons may significantly reduce patient morbidity and mortality [8,35]. Continuous postoperative monitoring of serum β-hCG levels until complete negativization is essential in follow-up. Given the rarity of these conditions, the establishment of multicenter registries and long-term clinical follow-up could contribute to improved understanding of their pathophysiology, optimization of diagnostic algorithms, and identification of potential risk factors that remain insufficiently recognized.

## 4. Conclusions

Our experience, when considered alongside the available literature, complements and reinforces several key clinical messages. OP and HP, although exceptionally rare forms of EP, should be included in the differential diagnosis in women of reproductive age presenting with abdominal pain and abnormal β-hCG dynamics to prevent severe complications that may threaten not only the patient’s life but also her future reproductive potential. Interestingly, in the first two cases presented in our series (Case 1 and Case 2), no classical risk factors for EP were identified. In contrast, the patient in the third case had a history of previous laparotomy and endometriosis, which may have contributed to the formation of adhesions and potentially facilitated this atypical site of implantation. The presented cases further emphasize the importance of timely recognition of atypical implantation sites of EP, as well as the need for a systematic diagnostic approach that includes serial monitoring of β-hCG levels and the appropriate use of modern imaging modalities. In situations where the diagnosis remains unclear, surgical exploration continues to represent a crucial method for establishing the definitive diagnosis and providing simultaneous treatment. Given the extreme rarity of these conditions, future multicenter studies and systematic data collection may contribute to a better understanding of their pathophysiology, optimization of diagnostic algorithms, and improvement of therapeutic strategies.

## Figures and Tables

**Figure 1:**
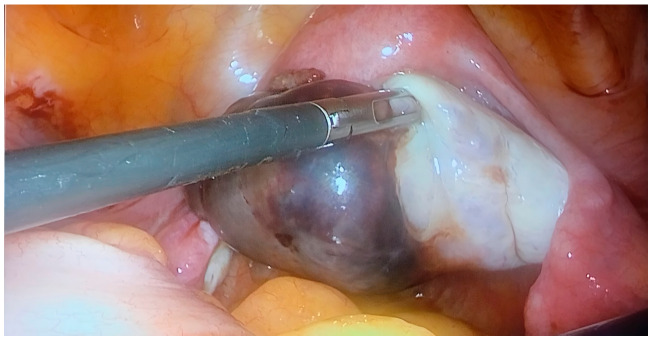
Laparoscopic view of the right ovary demonstrating an ectopic gestational mass consistent with ovarian pregnancy.

**Figure 2:**
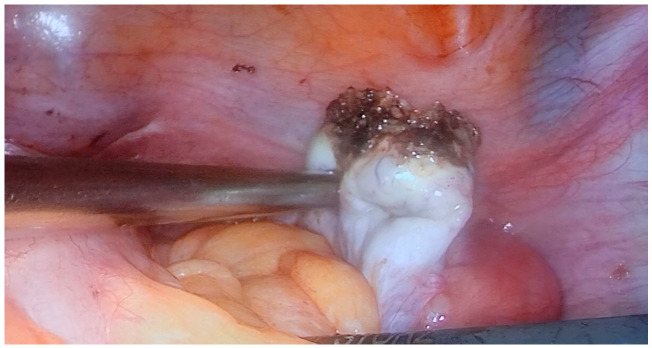
Intraoperative laparoscopic image showing partial ovariectomy performed for removal of the ectopic gestational tissue with preservation of the remaining ovarian parenchyma.

**Figure 3:**
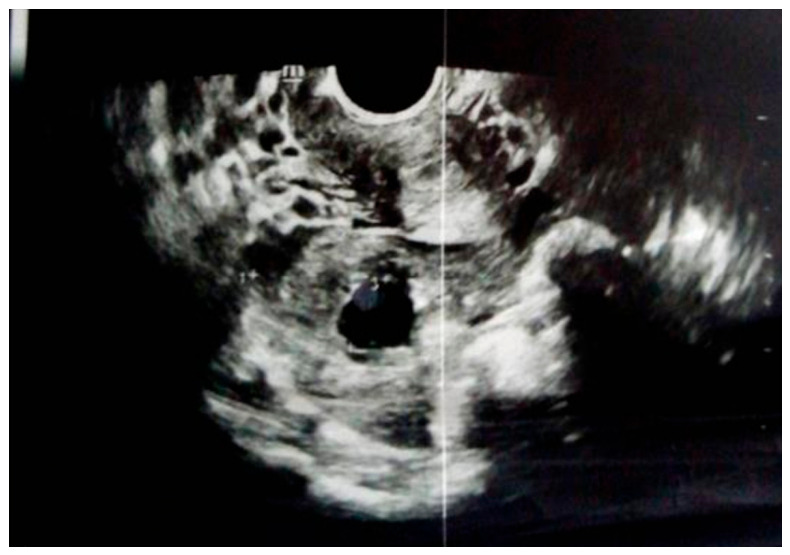
Transvaginal ultrasound image demonstrating a right adnexal mass with a cystic structure surrounded by echogenic ovarian tissue, suspicious for ovarian ectopic pregnancy. The uterus is retroverted with no visible intrauterine gestational sac. The lesion corresponds to the adnexal mass measuring 51 × 37 mm identified on TVUS.

**Figure 4:**
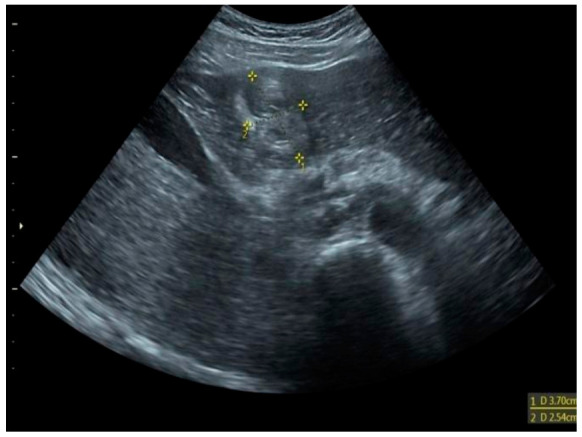
Abdominal ultrasound demonstrating a small focal hepatic lesion measuring approximately 37 × 25 mm (calipers shown).

**Figure 5:**
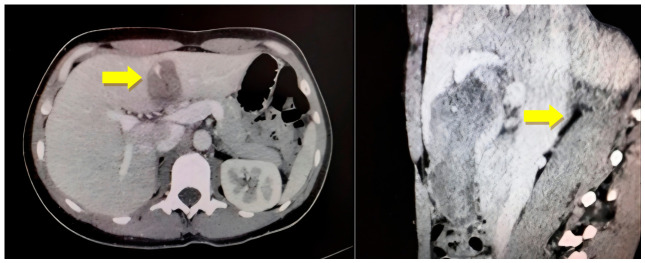
CT findings. (**Left**) Axial contrast-enhanced CT demonstrating an oval lesion within segment III of the liver measuring approximately 5 × 6 cm, characterized by central hypodensity with peripheral hyperdense enhancement (arrow). (**Right**) Coronal CT reconstruction confirms the hepatic lesion and associated blood collection located between the left hepatic lobe and the stomach (arrow).

**Figure 6:**
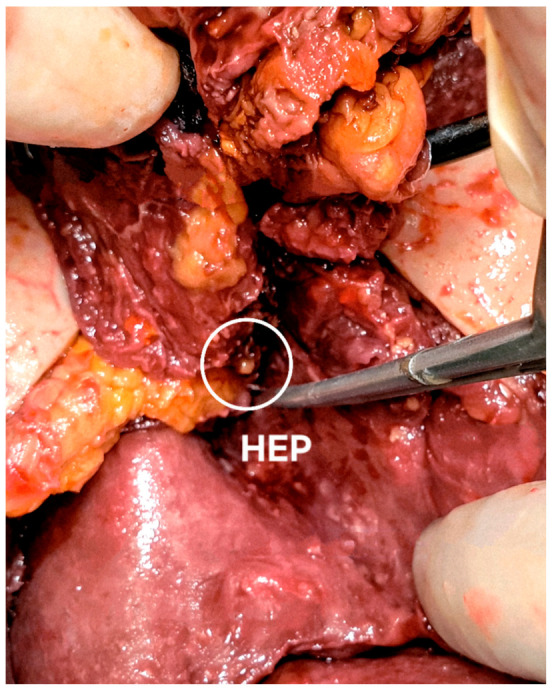
Intraoperative view demonstrating hepatic ectopic pregnancy (HEP) located on the surface of the left hepatic lobe, with associated hemoperitoneum and surrounding coagulated blood requiring surgical management.

**Table 1:** Comparative summary of clinical characteristics, diagnostic findings, and management of the three presented cases.

Parameter	Case 1	Case 2	Case 3
Age (years)	28	36	40
Gravidity/Parity	G0P0	G3P2	G0P0
Relevant medical history	Hypothyroidism, insulin resistance	Two previous cesarean deliveries, one miscarriage	Infertility; prior ovarian cystectomy due to endometriosis
Previous pelvic surgery	No	Cesarean sections	Pfannenstiel laparotomy for endometriosis
Gestational age at presentation	6 weeks	6 weeks	6 weeks
Main presenting symptom	Secondary amenorrhea	Pelvic pain and amenorrhea	Acute abdominal pain
Hemodynamic status	Stable	Stable	Hemodynamic instability
Initial β-hCG (mIU/mL)	~1134	18,773	621
Ultrasound findings	Right ovarian mass	Right adnexal mass with central anechoic area	Free intraperitoneal fluid; later hepatic lesion
Implantation site	Ovary	Ovary	Liver (segment III)
Surgical approach	Laparoscopy	Laparoscopy → adnexectomy	Laparotomy
Intraoperative findings	Ovarian ectopic pregnancy	Ruptured ovarian pregnancy with hemoperitoneum	Hepatic ectopic pregnancy with intra-abdominal bleeding
Estimated blood loss	Minimal	~400 mL	>2000 mL
Histopathological diagnosis	Ovarian ectopic pregnancy	Ovarian ectopic pregnancy	Hepatic ectopic pregnancy
Outcome	Uneventful recovery	Postoperative anemia, recovery	Uneventful recovery

Abbreviations: β-hCG, beta-human chorionic gonadotropin; G, gravidity; P, parity.

## Data Availability

The original contributions presented in this study are included in the article/Supplementary Material. Further inquiries can be directed to the corresponding author.

## Supplementary Materials

The following supporting information can be downloaded at https://www.mdpi.com/article/10.3390/clinpract16060107/s1: Table S1: Laboratory analyses of Case 1; Table S2: Laboratory analyses of Case 2; Table S3: Laboratory analyses of Case 3.

## Abbreviations

The following abbreviations are used in this manuscript:

EP	Ectopic Pregnancy
OP	Ovarian Pregnancy
HP	Hepatic Pregnancy
TVUS	Transvaginal Ultrasound
β-hCG	Beta-Human Chorionic Gonadotropin
ART	Assisted Reproductive Technologies
MTX	Methotrexate
US	Ultrasound
MRI	Magnetic Resonance Imaging
FDG PET-CT	Fluorodeoxyglucose Positron Emission Tomography–Computed Tomography
IUD	Intrauterine Device
CT	Computed Tomography
PID	Pelvic Inflammatory Disease
