# Supplementary material for: Rare Implantation Sites of Ectopic Pregnancy: A Case Series of Ovarian and Hepatic Pregnancy and Review of Diagnostic Challenges
Source: Clin Pract. 2026 May 31;16(6):107. doi: 10.3390/clinpract16060107 (PMC13297948; doi:10.3390/clinpract16060107)
Supplement: Supplementary file 1 [file clinpract-16-00107-s001.zip › suplementari s1.pdf]

Table S1. Laboratory analysis on admission day

Parameter	Value	Reference range
WBC (×10^9/L)	11.7	3.4–9.7
Neutrophils (%)	61.2	45–75
Lymphocytes (%)	29.6	20–45
Eosinophils (%)	1.5	0–5
Basophils (%)	0.6	0–1
Monocytes (%)	7.1	2–10
RBC (×10^{12}/L)	4.47	3.86–5.08
Hgb (g/L)	137	119–157
Plt (×10^9/L)	399	158–424
CRP (mg/L)	4.2	<5
Glucose (mmol/L)	5.1	4.1–6.1
ALT (U/L)	16	<34
AST (U/L)	13	<31

Abbreviations: WBC, white blood cells; RBC, red blood cells; Hgb, hemoglobin; Plt, platelets; CRP, C-reactive protein; ALT, alanine aminotransferase; AST, aspartate aminotransferase.
