# Supplementary material for: Rare Implantation Sites of Ectopic Pregnancy: A Case Series of Ovarian and Hepatic Pregnancy and Review of Diagnostic Challenges
Source: Clin Pract. 2026 May 31;16(6):107. doi: 10.3390/clinpract16060107 (PMC13297948; doi:10.3390/clinpract16060107)
Supplement: Supplementary file 1 [file clinpract-16-00107-s001.zip › suplementari s2.pdf]

Table S2. Laboratory analyses on admission day

Parameter	Value	Reference range
WBC ($\times 10^9$/L)	10.9	3.4–9.7
Neutrophils (%)	62.2	44–72
Lymphocytes (%)	29.7	20–46
Eosinophils (%)	1.6	0–7
Basophils (%)	0.5	0–1
Monocytes (%)	7.2	2–12
RBC ($\times 10^{12}$/L)	3.89	3.86–5.08
Hgb (g/L)	117	119–157
Plt ($\times 10^9$/L)	243	158–424
CRP (mg/L)	2.8	<5
Glucose (mmol/L)	4.8	4.1–6.1
ALT (U/L)	17	<35
AST (U/L)	14	<35

Abbreviations: WBC, white blood cells; RBC, red blood cells; Hgb, hemoglobin; Plt, platelets; CRP, C-reactive protein; ALT, alanine aminotransferase; AST, aspartate aminotransferase.
